# Self-harm and Suicidality Experiences of Middle-Age and Older Adults With vs. Without High Autistic Traits

**DOI:** 10.1007/s10803-022-05595-y

**Published:** 2022-05-26

**Authors:** Gavin R. Stewart, Anne Corbett, Clive Ballard, Byron Creese, Dag Aarsland, Adam Hampshire, Rebecca A. Charlton, Francesca Happé

**Affiliations:** 1 Social Genetic and Developmental Psychiatry Centre, Institute of Psychiatry, Psychology & Neuroscience, King’s College London, London, SE5 8AF UK; 2 College of Medicine and Health, University of Exeter, Exeter, EX1 2LU UK; 3 Department of Medicine, Imperial College London, London, SW7 2AZ UK; 4 Department of Psychology, Goldsmiths University of London, London, SE14 6NW UK

**Keywords:** Autistic traits, Older age, Self-harm, Suicidality, Suicide

## Abstract

Suicide has been identified as a leading cause of premature death in autistic populations. Elevated autistic traits have also been associated with higher rates of self-harm, suicidal ideation, and suicidal self-harm in the general population, but this has yet to be examined in older age. Using baseline cross-sectional data from the PROTECT study, middle-age and older adults with high autistic traits (n = 276) had significantly higher rates of suicidal ideation, deliberate self-harm, and suicidal self-harm than an age/sex-matched comparison group (n = 10,495). These differences represented a 5- to 6-fold increase in likelihood for self-harming and suicidality. These findings, which remained when controlling for depression symptoms, suggest that middle-age and older adults with high autistic traits may be particularly at risk of self-harm and suicidal behaviours.

**Supplementary Information:**

The online version contains supplementary material available at 10.1007/s10803-022-05595-y.

## Introduction

Autism represents a highly heritable heterogenous group of lifelong neurodevelopmental conditions, characterised by differences in social communication and restricted-repetitive behaviours (American Psychiatric Association, 2013). Autism and autistic traits have been associated with a wide range of negative outcomes across the lifespan, with a recent meta-analysis reporting that approximately half of all autistic people have ‘poor’ life outcomes (Mason et al., 2020), such as lower education attainment and employment rates and negative social outcomes than non-autistic populations (Steinhausen et al., 2016). Across the adult lifespan, people on the autism spectrum, i.e. those who either meet diagnostic thresholds or who endorse high autistic traits, also often report poorer physical and mental health (Croen et al., 2015; Lever & Geurts, 2016; Stewart et al., 2020a), higher rates of traumatic life events (Rumball et al., 2020; Stewart et al., 2022), more sleep disruptions (Richdale & Schreck, 2009; Stewart et al., 2020b), and lower quality of life than the general population (Ayres et al., 2017; Mason et al., 2019b). Despite these negative outcomes and experiences and their associated risk for periods of crisis, self-harm and suicidality have only recently been studied in autistic populations (Cassidy & Rodgers, 2017).

Several studies—including those that use large population registers and genetically informed designs—have now documented that autistic and high autistic trait populations are at increased risk for suicidal ideation (Cassidy et al., 2014; Raja, 2011; Warrier & Baron-Cohen, 2019), non-suicidal self-harm (Cassidy et al., 2018b; Maddox et al., 2017; Raja, 2011; Warrier & Baron-Cohen, 2019), suicidal self-harm (Cassidy et al., 2018a, 2018b; Hirvikoski et al., 2020; Pelton et al., 2020; Warrier & Baron-Cohen, 2019), and death by suicide (Cassidy et al., 2022; Hirvikoski et al., 2016, 2020; Hwang et al., 2019; Kirby et al., 2019). Autistic adults without intellectual disabilities (ID) have been found to be at an almost a tenfold risk increase for death by suicide than non-autistic people using Swedish population registers (OR 9.40 [7.42–11.90]; Hirvikoski et al., 2016). This high rate of death by suicide in people with evidence of autism has also been found in English coroner inquest reports (OR 11.08 [3.92–31.31]; Cassidy et al., 2022). Furthermore, injury and poisoning (which includes self-harm and suicide) have also been identified as one of the most common causes of death for autistic people (with and without ID) in an Australian population register (Hwang et al., 2019). The findings from these population registers suggest that suicide is a leading cause of premature death among autistic people without ID (Hirvikoski et al., 2016; Hwang et al., 2019), and that high autistic traits are significantly over-represented in those who die by suicide (Cassidy et al., 2022). Furthermore, while non-autistic men are more likely than women to self-harm and die by suicide in the general population (Office for National Statistics, 2019), the pattern is somewhat more complex in autistic populations. Autistic women without ID have been found to be at a higher risk for suicidal self-harm resulting in inpatient treatment than autistic men without ID (11.63% vs. 4.18%), but similar rates of death by suicide are found (0.52% vs. 0.62%) (Hirvikoski et al., 2020). Despite this, findings from Hirvikoski et al. (2016) document that autistic women are still at a far higher likelihood to die by suicide than non-autistic women (0.3% vs. 0.03%; OR 13.05 [8.73–19.50]), as well as autistic men compared to non-autistic men, albeit to a lesser extent (0.3% vs. 0.07%; OR 6.28 [4.79–8.23]). A similar pattern of results is also found in (Hirvikoski et al., 2020). These high rates of suicidality have also been found in populations that endorse high autistic traits (e.g., Cassidy et al., 2022; South et al., 2020). Suicidality, including rates of self-harming behaviours, have been found to increase with autistic traits (Cassidy et al., 2018a, 2018b; Pelton & Cassidy, 2017), and are also experienced at increased rates by first-degree relatives of autistic people (Hirvikoski et al., 2020).

It is important to note that while self-harm is a significant risk factor for suicidal behaviours, many autistic and non-autistic people self-harm for non-suicidal reasons. Prevalence estimates suggest that approximately 50% of autistic people and 20% of non-autistic people have self-harmed for non-suicidal reasons (Cassidy et al., 2018a, 2018b, 2020a, 2020b; Maddox et al., 2017; Whitlock et al., 2014). While the majority of people who engage in non-suicidal self-harm do not go on to experience suicidal self-harm, theories of suicide place non-suicidal self-harm as a key precursor to the future development of suicidal behaviours (Cassidy et al., 2018a, 2018b; Mars et al., 2019; Pelton et al., 2020). Many factors have been found to contribute to the experience of suicidality in autistic and high autistic trait populations, such as persistent symptoms of depression (South et al., 2020), traumatic experiences (Warrier & Baron-Cohen, 2019), thwarted belonging and perceived burdensomeness (Pelton & Cassidy, 2017; Pelton et al., 2020), poor sleep quality (Hochard et al., 2020), and as a consequence of camouflaging autistic behaviours in social situations (Cassidy et al., 2020a, 2020b). Many of these experiences have previously been documented at high rates in those on the autism spectrum across the lifespan.

While risk for suicide fluctuates across the lifespan (Office for National Statistics, 2019), much of the self-harm and suicidality literature to date has focused on young to middle-age autistic adults. As autism affects ~ 1% of the population (Brugha et al., 2016), there are an estimated 650,000 autistic people in the UK, with approximately 240,000 of these individuals being over 50 years of age (Brugha et al., 2016; Office for National Statistics, 2018). While self-harm and suicidal ideation are more common in younger populations, adults aged 45–64 in the general population are found to be in the highest risk group for death by suicide (Office for National Statistics, 2019; Troya et al., 2019). A recent systematic review on self-harm and suicidality in older age noted that older adults who have self-harmed are 145 times more likely to die by suicide than those who have not (Troya et al., 2019). However, self-harm and suicidality have yet to be studied in older adults on the autism spectrum. Despite this need for further research, there are several challenges and barriers to the study of ageing in autism. The first cohort of children diagnosed in the 1960s is only now entering older age. Additionally, changes to diagnostic criteria over the past 60 years also mean those early diagnosed individuals are not representative of adults diagnosed today (Lai & Baron-Cohen, 2015; Stuart-Hamilton et al., 2010). Furthermore, many adults who self-identify as autistic face significant barriers when trying to access assessments for a formal diagnosis (Lewis, 2017). This is partly due to autism being the purview of child psychiatrists, thus adult services often overlook autism as a later life diagnosis resulting in many autistic older adults remaining undiagnosed, being labelled ‘the lost generation’ (Brugha et al., 2016; Lai & Baron-Cohen, 2015; Office for National Statistics, 2018; Stuart-Hamilton et al., 2010). Autism is also now increasingly seen as lying at the end of a dimension of socio-communicative difficulties, with overlapping genetic influences operating on diagnosed autism and sub-clinical autistic traits in the general population (Bralten et al., 2018; Lundström, 2012; Whitehouse et al., 2011). Research taking a dimensional, trait-wise approach to autism is becoming increasingly common as this increases statistical power by including a large number of individuals with high levels of autistic characteristics who nonetheless fall below the diagnostic threshold (or who lack a formal diagnosis). This approach may be particularly useful for exploring autism-related issues in under-studied and under-diagnosed groups, such as older adults (Mason et al., 2022). As support needs often change with age, knowledge about the experiences of self-harm and suicidality in middle-aged and older adults on the autism spectrum could give indications about the support needs for this large and growing population (Stuart-Hamilton et al., 2010).

The current study investigates the self-reported prevalence of self-harming behaviours and suicidal thoughts and behaviours in a large sample of adults age 50 years plus. It is hypothesised that older adults with high autistic traits will self-report (1) more suicidal ideation, (2) more self-harming thoughts, (3) more deliberate self-harming incidents, and (4) more suicidal self-harm incidents than an age- and sex-matched low autistic trait comparison group. It is also hypothesised that (6) the higher rates of self-harm and suicidality reported by those with high autistic traits (vs. the comparison group) will not be entirely accounted for by higher current depression symptoms.

## Methods

### Study Design

This study uses cross-sectional baseline data from the PROTECT study (www.protectstudy.org.uk). Inclusion criteria for the PROTECT study are: aged over 50 years, resident in the UK, with good working understanding of English, and able to use a computer with internet access. Participants who have an established diagnosis of dementia are excluded. Participants register online and are required to review the study information sheet and to provide informed consent via an approved online platform. The PROTECT study received ethical approval from the UK London Bridge National Research Ethics Committee (Ref: 13/LO/1578).

### Participants

From a total sample of 20,220 participants (female n = 14,946, 73.9%), 276 (1.4%) met our cut-off criteria for the Autism Spectrum Traits (AST) group; see Measures section below for inclusion criteria. To create a Control Older Adults (COA) group, from the remaining 19,944 participants, 4243 participants were excluded for endorsing any autism spectrum traits. To match the AST and COA groups on mean age/range and sex ratio, a further 5206 participants were excluded using random participant selection methods, resulting in 10,495 participants in the COA group. To ensure that the COA group selected was not unrepresentative of the PROTECT cohort as a whole, analyses were repeated comparing the AST group to all other PROTECT participants (n = 19,944); the same pattern of results was found as when comparing AST with COA, suggesting that the COA sample selected for no autistic traits was not unrepresentative or atypical. See Table 1 for demographic characteristics and Supplementary Materials Table 1 for demographic characteristics split by sex.

**Table 1:** Demographic characteristics and depression symptoms of COA and AST groups

	Control older adults (COA; n = 10,495)		AS traits (AST; n = 276)		Group difference	Effect size (Cohen's *d*)	Odds ratio
Age (years)							
M (SD)	62.42	(6.67)	62.97	(6.74)	F(1,10,769) = 1.73, *p* = 0.176	0.08 [− 0.04 to 0.01]	–
[95% CI]	[62.29–62.55]		[62.17–63.77]				
Range	50–81		50–81				
Sex							
N male:female	3200:7295		90:186		χ^2^ = 0.57, *p* = 0.451	0.05 [− 0.09 to 0.19]	–
%	30.5%:69.5%		32.6%:67.4%				
Marital status							
Married	7517	(71.8%)	171	(62.0%)	χ^2^ = 22.78, *p* < 0.001***	0.27 [0.15–0.39]	–
Widowed	476	(4.5%)	8	(2.9%)			
Separated	177	(1.7%)	5	(1.8%)			
Divorced	990	(9.5%)	37	(13.4%)			
Civil partnership	56	(0.5%)	1	(0.4%)			
Co-habiting	684	(6.5%)	28	(10.1%)			
Single	570	(5.4%)	26	(9.4%)			
Education history							
School to 16	1444	(13.8%)	43	(15.6%)	χ^2^ = 2.97, *p* = 0.395	0.02 [-0.10–0.14]	–
School to 18	3264	(31.2%)	73	(26.4%)			
Undergraduate	3566	(34.1%)	99	(35.9%)			
Postgraduate	2196	(21.0%)	61	(22.1%)			
Current employment status							
Employed	5809	(55.5%)	140	(50.7%)	χ^2^ = 4.72, *p* = 0.094	0.12 [0.01–0.23]	–
Retired	4331	(41.4%)	122	(44.2%)			
Unemployed	330	(3.2%)	14	(5.1%)			
Autism diagnosis							
% Yes	0	–	21	(7.6%)	χ^2^ = 800.09, *p* < 0.001	1.79 [1.67–1.92]	–
Depression (max score = 24)							
M (SD)	2.24	(2.77)	6.05	(4.94)	F(1,10,766) = 480.05, *p* < 0.001***	1.33 [1.21–1.45]	–
[95% CI]	[2.19–2.30]		[5.46–6.63]				
N (%) above cut-off	300	(2.9%)	58	(21.0%)	χ^2^ = 275.78, *p* < 0.001***	1.21 [1.04–1.38]	9.04 [6.61–12.34]

Some sex differences were found in age, martial status, education history, and current employment status. See Supplementary Materials Table 1 for demographics split by sex

*p < 0.05, **p < 0.01, ***p < 0.001

Age and sex ratio (i.e. matched characteristics), as well as education history and employment status, did not differ between the AST and COA groups. Differences between the AST and COA groups were observed in marital status, with AST more often being divorced, co-habiting, or single, and COA more often being married or widowed.

### Measures

Demographic information was collected using PROTECT’s online survey platform, including age, sex, marital status, education history, and employment status.

Autistic traits were measured using the PROTECT Autism Spectrum Traits questionnaire (Stewart et al., 2020a). The questionnaire comprises five yes/no items, asking about childhood (n = 2) and current (n = 3) socio-communicative autistic traits. Participants who endorsed both childhood traits plus at least two of the three current traits met criteria for the AST group. Those in the COA group did not endorse any traits. In a separate sample, the questionnaire showed good internal consistency (Cronbach’s *a* = 0.82), sensitivity (82%) and specificity (94%) for identifying those with an autism diagnosis (Stewart et al., 2020a).

Experiences of self-harm and suicidality were measured using a set of questions adapted for use in PROTECT from the UK Biobank test package. The 7-item questionnaire in PROTECT asked participants if they (1) have thought life was not worth living or (2) have ever contemplated harming themselves. Further questions ask if they (3) have ever deliberately harmed themselves, and if so, (4) how many times they have harmed themselves and (5) what types of self-harming behaviours they have used. Participants are also asked if they (6) have harmed themselves with the intention of ending their lives. Participants who have self-harmed are also asked whether they (7) have sought help or support after a self-harming (whether suicidal or non-suicidal) incident. These questions are scored on either a yes/no scale or frequency scale. See Supplementary Materials for full question list and scoring matrix.

Symptoms of recent depression were measured using the Patient Health Questionnaire (PHQ-8; (Kroenke et al., 2009). The PHQ-8 is an eight-item questionnaire (rated on a 4-point scale, maximum score = 24) examining low mood over the past two weeks. The PHQ-8 is adapted from the original nine-item PHQ, with the omission of the self-harm and suicidality item. The PHQ-8 was used to avoid item overlap with the PROTECT self-harm and suicidality questionnaire. The PHQ-8 has excellent psychometric properties, with a cut-off score of ≥ 10 having 88% sensitivity and 88% specificity for major depression (Kroenke et al., 2001, 2009). The original nine-item PHQ has been found to have good psychometric properties for assessing depression symptoms in autistic populations (Cassidy et al., 2018a, 2018b).

### Statistical Analyses

All statistical analyses were performed using SPSS (version 25.0; IBM Corp., 2017). Differences between AST and COA in demographic variables were analysed using analysis of variance (ANOVA) and chi-square (χ^2^) tests. χ^2^ was also used to evaluate differences in self-harm and suicidality questionnaire responses. To account for the influence of depressive symptoms on self-harm and suicidality in the AST and COA groups, additional comparisons were made between those with low vs. high current symptoms of depression. Additional ANOVA and χ^2^ analyses were also conducted to examine sex differences. Associations with age were also examined using correlation analyses, with Fisher’s r-to-z transformations being conducted to compare correlation coefficients. Multiple comparisons were controlled for using the False Discovery Rate (FDR) method (Benjamini & Hochberg, 1995), with an initial α-value of 0.05 being used. FDR was applied to all *p*-values, with adjusted α-values being assigned based on the *p*-value rank.

## Results

### Suicidal Ideation and Thoughts of Self-harm

**Fig. 1:**
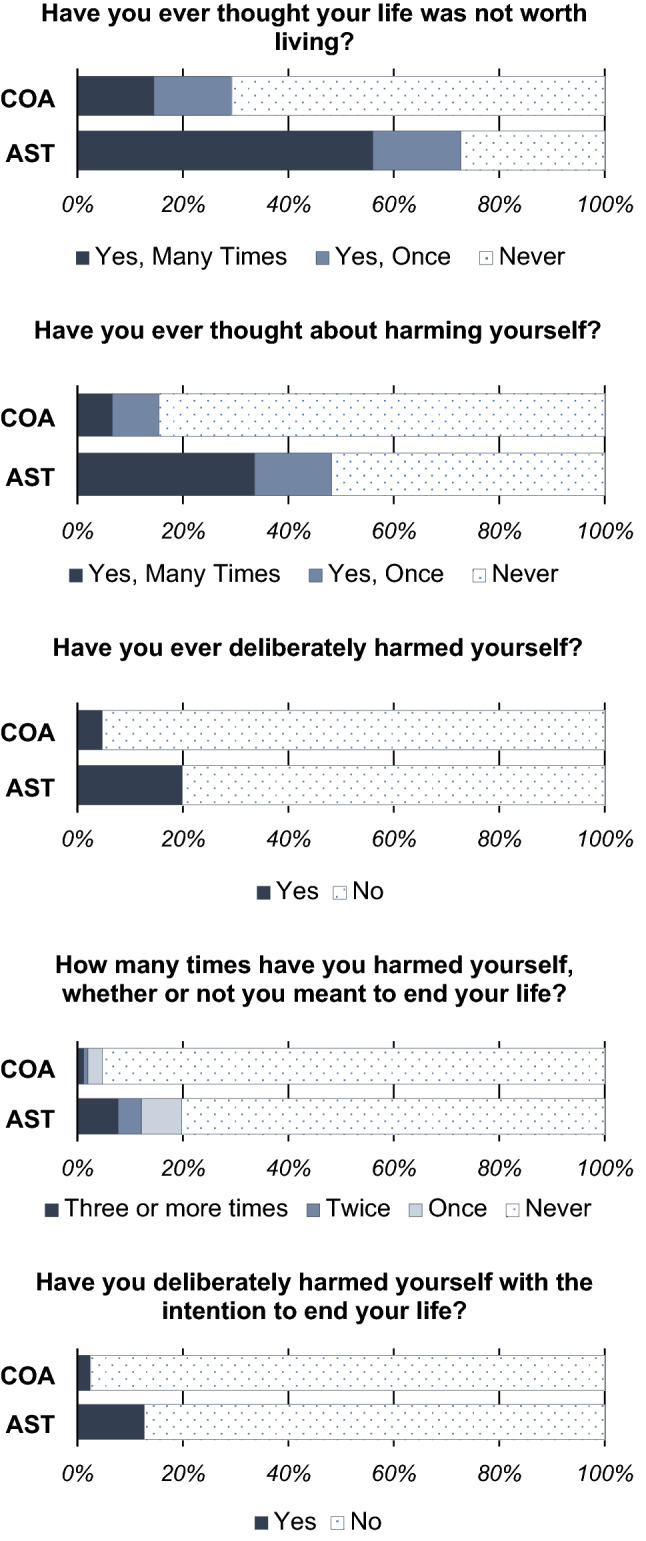
Rates of suicidal ideation, self-harm, and suicidal self-harm

The AST group had significantly higher frequencies of suicidal ideation (‘*Many people have thoughts that life is not worth living. Have you felt that way?’)* and thoughts of self-harm *(‘Have you contemplated harming yourself?’)* than the COA group. Approximately 73% of the AST group had experienced suicidal ideation and thoughts of self-harm compared to only 30% of the COA group. These differences were of large effect size (Cohen’s *d* = 1.06–1.15). The AST group were at a sixfold likelihood for suicidal ideation (OR 6.43 [4.91–8.41]), and a fivefold likelihood for thoughts of self-harm compared to the COA group (OR 5.10 [4.00–6.50]). See Table 2 and Fig. 1 for prevalence rates of self-harm and suicidality thoughts/behaviours.

**Table 2:** Self-reported prevalence rates of self-harm, suicidal ideation and behaviour in COA and AST groups

	Control older adults (COA; n = 10,495)		AS traits (AST; n = 276)		Group difference	Effect size (Cohen's *d*)	Odds ratio
Many people have thoughts that life is not worth living. Have you felt that way?							
No	7372	(70.7%)	75	(27.3%)	χ^2^ = 369.14 *p* < 0.001***	1.15 [1.03–1.27]	6.43^ǂ^ [4.91–8.41]
Yes, once	1543	(14.8%)	46	(16.7%)			
Yes, more than once	1514	(14.5%)	154	(56.0%)			
Have you contemplated harming yourself? E.g. by cutting, biting, hitting yourself, taking an overdose							
No	8843	(84.6%)	142	(51.8%)	χ^2^ = 313.22 *p* < 0.001***	1.06 [0.94–1.18]	5.10^ǂ^ [4.00–6.50]
Yes, once	926	(8.9%)	40	(14.6%)			
Yes, more than once	685	(6.6%)	92	(33.6%)			
Have you deliberately harmed yourself whether or not you meant to end your life?							
No	9959	(95.3%)	218	(80.1%)	χ^2^ = 125.83 *p* < 0.001***	0.69 [0.57–0.81]	5.01 [3.67–6.85]
Yes	492	(4.7%)	54	(19.9%)			
How many times have you harmed yourself?							
Never	10,003	(95.5%)	222	(80.7%)	χ^2^ = 149.73 *p* < 0.001***	0.75 [0.63–0.87]	5.03^ǂ^ [3.68–6.89]
Once	266	(2.5%)	20	(7.3%)			
Twice	88	(0.8%)	12	(4.4%)			
Three or more times	121	(1.2%)	21	(7.6%)			
Have you deliberately harmed yourself with the intention to end your life?							
No	10,226	(97.5%)	241	(87.3%)	χ^2^ = 103.42 *p* < 0.001***	0.62 [0.50–0.74]	5.65 [3.88–8.22]
Yes	263	(2.5%)	35	(12.7%)			

Self-harm and suicidal thoughts and behaviours measured using a bespoke measure

^ǂ^Odds ratio calculated with yes/frequency options collapsed

*p < 0.05, **p < 0.01, ***p < 0.001

### Deliberate Self-harm and the Methods Used

The AST group reported significantly higher frequencies of deliberate self-harming behaviours (*‘Have you deliberately harmed yourself whether or not you meant to end your life?’)*, as well as a higher frequency of self-harming incidents (*‘How many times have you harmed yourself?’)* than the COA group. Approximately 20% of the AST group had deliberately self-harmed compared to only 5% in the comparison group. These differences had moderate-to-large effect sizes (Cohen’s *d* = 0.69–0.75). The AST group were at a fivefold likelihood for deliberately self-harming compared to the COA group (OR 5.01 [3.68–6.89]). See Table 2 and Fig. 1 for prevalence rates of self-harm and suicidality thoughts/behaviours.

When considering the overlap between suicidal ideation and thoughts of self-harm with deliberate self-harm, 25% of the AST group who experienced suicidal ideation and thoughts of self-harm also deliberately self-harmed, compared to 14% in the COA group. This difference was significant (χ^2^ = 19.96, *p* < 0.001) with a moderate effect size (Cohen’s *d* = 0.41).

Additionally, the AST group reported significantly higher rates than COA for all the self-harming methods examined, including (1) self-injury such as cutting/scratching/hitting, (2) ingesting medication in excess of normal dosage, (3) abusing alcohol or non-prescription drugs, (4) swallowing dangerous objects/substances, and (5) stopping prescribed medications without medical supervision. Furthermore, the AST group were more likely to use multiple self-harming methods compared to COA. These differences were of moderate-to-large effect size (Cohen’s *d* = 0.71–1.35). For self-injury and ingesting medication in excess of normal dosages, the AST group were at a four-to-fivefold likelihood of these self-harming behaviours compared to the COA group. See Table 3 for list of self-harm methods used and the total number of methods used, and for individual odds ratios and confidence intervals.

**Table 3:** Frequency of types of self-harm behaviours reported by COA and AST groups

	Control older adults (COA; n = 10,495)		AS traits (AST; n = 276)		Group difference	Effect size (Cohen's *d*)	Odds ratio
Type of self-harm behaviours							
Self-injury (e.g. cutting, scratching, hitting)	240	(2.3%)	24	(8.7%)	χ^2^ = 46.46, *p* < 0.001***	0.77 [0.53–1.01]	4.08 [2.64–6.33]
Ingesting medication in excess of the normal dose	281	(2.7%)	35	(12.7%)	χ^2^ = 94.99, *p* < 0.001***	0.91 [0.71–1.12]	5.29 [3.65–7.70]
Abusing alcohol or non-prescription drugs	57	(0.5%)	11	(4.0%)	χ^2^ = 51.02, *p* < 0.001***	1.11 [0.75–1.48]	7.63 [3.95–14.71]
Swallowing dangerous objects/substances	8	(0.1%)	2	(0.7%)	χ^2^ = 12.24, *p* < 0.001***	1.24 [0.38–2.21]	9.60 [2.03–45.42]
Stopping prescribed medication without consulting a medical professional	20	(0.2%)	6	(2.2%)	χ^2^ = 44.11, *p* < 0.001***	1.35 [0.85–1.86]	11.68 [4.65–29.32]
Total number of self-harm behaviours used							
None	10,024	(95.5%)	227	(82.5%)	χ^2^ = 147.95, *p* < 0.001***	0.71 [0.59–0.83]	-
One behaviour	367	(3.5%)	26	(9.5%)			
Two behaviours	69	(0.7%)	16	(5.8%)			
Three or more behaviours	32	(0.3%)	6	(2.2%)			

*p < 0.05, **p < 0.01, ***p < 0.001

### Suicidal Self-harm

The AST group reported significantly higher rates of suicidal self-harm *(‘Have you deliberately harmed yourself with the intention to end your life?’)* than the COA group. Approximately 13% of the AST group had experienced suicidal self-harm compared to only 3% of the comparison group. This difference had a moderate effect size (Cohen’s *d* = 0.62). The AST group were at a near sixfold likelihood for suicidal self-harm compared to the COA group (OR 5.65 [3.88–8.22]). See Table 2 and Fig. 1 for prevalence rates of self-harm and suicidality.

When considering the overlap of suicidal ideation to suicidal self-harm, 18% of the AST group who experienced suicidal ideation also experienced suicidal self-harm, compared to 8% in the COA group. This difference was significant (χ^2^ = 20.72, *p* < 0.001) with a moderate effect size (Cohen’s *d* = 0.48). Furthermore, for the overlap of deliberate self-harm to suicidal self-harm, 64% of the AST group who deliberately self-harmed also experience suicidal self-harm, compared to 53% in the COA group. This difference was not significant (χ^2^ = 2.25, *p* = 0.134).

#### Seeking Help After Deliberate Self-harm and/or Suicidal Self-harm Incidents

The AST and COA groups had similar rates of help seeking behaviors after self-harming or suicidal incidents. These similarities were found in the rates of seeking any treatment, hospital treatment, support from friends and family, or contacting a helpline. However, the AST group were significantly more likely than the COA group to seek help from a psychiatrist (57% vs. 31%), or their GP (34% vs. 21%). These differences had small-to-moderate effect sizes (Cohen’s *d* = 0.34–0.57). See Table 4 for frequency of help-seeking behaviours.

**Table 4:** Self-report frequencies of support sought after a self-harm incident in the COA and AST groups

	Control older adults (COA; n = 492)		AS traits (AST; n = 53)		Group difference	Effect size (Cohen's *d*)
Following any time when you took an overdose or deliberately tried to harm yourself did you seek…						
Any treatment?	313	(63.6%)	40	(75.5%)	χ^2^ = 2.95 *p* = 0.086	0.27 [− 0.07 to 0.62]
Hospital treatment?	219	(44.5%)	31	(58.5%)	χ^2^ = 3.76 *p* = 0.052	0.28 [− 0.03 to 0.59]
Psychiatric treatment?	152	(30.9%)	30	(56.6%)	χ^2^ = 14.22 *p* < 0.001***	0.57 [0.25–0.88]
GP support?	104	(21.1%)	18	(34.0%)	χ^2^ = 4.53 *p* = 0.033*	0.34 [0.01–0.68]
Support from family/friends?	159	(32.3%)	14	(26.4%)	χ^2^ = 0.67 *p* = 0.380	− 0.17 [− 0.52 to 0.18]
Helpline support?	41	(8.3%)	7	(13.2%)	χ^2^ = 1.42 *p* = 0.234	0.27 [− 0.19 to 0.74]

*p < 0.05, **p < 0.01, ***p < 0.001

#### Influence of Current Symptoms of Depression

The AST group reported significantly more symptoms of current depression than the COA group. Additionally, more individuals in the AST group passed the cut-off score for probable clinical levels of depression, compared to the COA group. These differences had large effect sizes (Cohen’s *d* = 1.21–1.33). See Table 1 for depression symptom score and cut-off summary.

To establish whether the higher rates of self-harm and suicidality in the AST group were solely a function of their worse depression symptomatology, the analyses were repeated taking account of likely depression status. Elevated rates of all self-harm and suicidality behaviours were found in the AST group vs the COA group when examining only those below the cut-off for probable clinical levels of depression on the PHQ-8 (ORs 4.5–5.8). Additionally, the AST group below PHQ-8 cut-off still reported higher current depression scores than the COA group with a large effect size (Cohen’s *d* = 0.96). The same pattern of results was also found when examining only those above the cut-off for probable clinical levels of depression (ORs 1.9–3.0), including in current depression symptoms to a moderate effect size (Cohen’s *d* = 0.39). See Supplementary Materials Tables 2 and 3 for prevalence rates of self-harm and suicidality behaviours split by AST/COA and above/below depression cut-off groups, and for individual odds ratios and confidence intervals.

#### Association of Self-harm and Suicidality Behaviours with Age

In the AST group, some significant small negative associations were found between self-harm and suicidality behaviours and age; older age was associated with lower rates of contemplating self-harm (*r* =  − 0.247, *p* < 0.001), deliberate self-harm (*r* =  − 0.183, *p* = 0.002), the number of times self-harmed (*r* =  − 0.158, *p* = 0.008), and suicidal self-harm (*r* =  − 0.176, *p* = 0.003). No other significant associations with age were found.

In the COA group, significant but very weak negative associations were found between self-harm and suicidality behaviours and age; older age was associated with lower rates of suicidal ideation (*r* =  − 0.092, *p* < 0.001), contemplating self-harm (*r* =  − 0.100, *p* < 0.001), deliberate self-harm (*r* =  − 0.067, *p* < 0.001), the number of times self-harmed (*r* =  − 0.068, *p* < 0.001), and suicidal self-harm (*r* =  − 0.035, *p* < 0.001).

Using Fisher’s r-to-z transformation, the strength of association with age was significantly greater in the AST group than COA for contemplating self-harm (z =  − 2.45, *p* = 0.007), deliberate self-harm (z =  − 1.92, *p* = 0.027), and suicidal self-harm (z =  − 2.33, *p* = 0.010) meaning the older COA participants were less likely to show these behaviours than the older AST participants. No other differences were found in association strengths.

#### Sex Differences in Prevalence Rates

No sex differences were found in the AST group for any of the analyses. COA women had significantly higher rates of suicidal ideation, thoughts of self-harm, deliberate self-harm, and suicidal self-harm, when compared to COA men.

## Discussion

The current study documents for the first time the prevalence of self-reported self-harm and suicidality in a large sample of middle-aged and older adults with (compared to without) high autistic traits. In keeping with the previous literature that examines younger and middle-aged autistic adults, the adults with high socio-communicative autistic traits in the current study reported significantly higher rates of suicidal ideation, deliberate self-harm, and suicidal self-harm than comparison adults with low autistic traits. These findings remained when taking into account the higher levels of depression symptoms reported by the high autism traits group. Furthermore, while the majority of people who experienced suicidal ideation did not also experience suicidal self-harm, significantly more people with high versus low autistic traits did so. However, help-seeking behaviours after self-harm were comparable in both groups, with the majority of those who did self-harm going on to seek support from a healthcare professional, friends and family, or a support helpline. While low autistic trait women had higher rates of self-harm and suicidality than low autistic trait men, this difference was not found in the high autistic traits group. These results suggest that the high rates of self-harm and suicidality previously documented in younger autistic populations are also found in older age in those with high autistic traits. As there is an age-related peak in suicidality risk in people age 45–60 in the general population (Office for National Statistics, 2019), middle-age and older adults with high autistic traits could be at an increased susceptibility to experience self-harm and suicidality.

The first key finding in the current study is that middle-aged and older adults with high autistic traits reported higher rates of suicidal ideation and thoughts of self-harm compared to those with low autistic traits. This study documented that 73% of the older high autistic trait adults experienced suicidal ideation; this finding is consistent with the previous literature that examines younger and middle-aged autistic adults; Cassidy et al. (2014) document that 66% of their autistic young to middle-aged adult sample that had been recently diagnosed with Asperger’s syndrome at a specialist UK-based diagnostic clinic had experienced suicidal ideation. Additionally, Arwert and Sizoo (2020) also document that 67% of their autistic young to middle-aged adult sample recruited through Dutch outpatient healthcare services for autistic adults’ self-report experiences of suicidal ideation. These rates are comparable and suggest that the high rates of suicidal ideation documented in younger autistic populations are also found in high autistic trait populations in older age. However, it is important to note that these two studies involve autistic adults (rather than high autistic trait adults) and their samples are recruited through clinics, rather than being an online survey. Furthermore, prevalence estimates using National Health Service records suggest that suicidal ideation is somewhat common in the general population; approximately 20% of adults experience suicidal thoughts at some point in their lifetime (McManus et al., 2016). However, the rates found in the general population compared to the rates in the current study and Cassidy et al. (2014) suggest that people on the autism spectrum may be at a three-to-fourfold likelihood of experiencing suicidal ideation compared to the general population.

When considering why autistic people have such a high likelihood of suicidality, it is important to consider commonly co-occurring health problems and experiences. Suicidal ideation is often associated with mental health difficulties (Cassidy et al., 2018a, 2018b). Autistic populations, including middle-aged and older autistic adults, experience almost all mental health conditions at increased rates compared to non-autistic comparison groups (Bishop-Fitzpatrick & Rubenstein, 2019; Croen et al., 2015; Lever & Geurts, 2016). These high rates of mental health problems have also been documented in the high autistic trait sample described in this study (Stewart et al., 2020a, 2020b, 2022). However, while co-occurring conditions such as depression may increase the likelihood of experiencing suicidality, in the current study the high autism traits group showed high rates of suicidality even after accounting for (stratifying by) level of depression symptoms. Additionally, experiences such as autistic burnout may play an important role, with some recent qualitative studies providing anecdotal evidence of burnout being linked to suicidality (Higgins et al., 2021; Raymaker et al., 2020). Other issues, such as having unmet support needs and camouflaging autistic behaviours may also contribute to the experience of mental health crises, resulting in increased rates of suicidality (Cassidy et al., 2018a, 2018b; Turcotte et al., 2016). Further research is needed that examines the relationship between these experiences and suicidality in autistic populations, particularly studies that can disentangle the difference between past and current suicidal ideation/behaviours from mental health difficulties. Furthermore, research that can examine the mechanisms underpinning the escalation of these mental health difficulties to periods of suicidal ideation is needed to understand why people on the autism spectrum experience these high rates of suicidality.

The second key finding in the current study is that middle-aged and older adults with high autistic traits report higher rates of deliberate self-harm compared to those with low autistic traits (20% vs 5%). Additionally, the high autistic trait group were also more likely to self-harm more frequently, and to report a wider range of self-harming methods than the comparison group (e.g., self-injury, overdosing, abusing hazardous substances). Furthermore, a higher rate of progression from suicidal ideation to deliberate self-harm was found in the high autistic traits group compared to the comparison group (25% vs. 14%). These higher rates of self-harm were found to persist when taking into account severity of current depression symptoms. While the phrasing of the deliberate self-harm question used in this study does not differentiate the motive of the self-harm (i.e., non-suicidal self-harm or suicidal self-harm), the rates documented in the current study are lower than the self-harm rates reported in studies, e.g., 50–65% of autistic people reporting non-suicidal self-harm in Cassidy et al., (2018a, 2018b) and Maddox et al. (2017). These differences in prevalence could be due to sampling biases and recruitment differences between studies. For example, Cassidy et al., (2018a, 2018b) and Maddox et al. (2017) recruited autistic participants to studies about mental health, self-harm and suicidality, whereas PROTECT is a healthy ageing study that uses a comprehensive set of questionnaires about health, cognition, social functioning, and psychopathology (including self-harm and suicidality). Despite the prevalence differences, our results still suggest that people on the autism spectrum, including those with high autistic traits, experience self-harming behaviours at greatly increased rates compared to the general population. This could be particularly important for autistic women; while no sex differences were found in the current study, autistic women have been found to experience non-suicidal self-harm at higher rates than autistic men in other studies [e.g., Cassidy et al., (2018a, 2018b)]. As non-suicidal self-harm is an important predictor of later suicidal self-harm, specialised support and interventions targeting autistic people who experience self-harm could address the high rates of death by suicide in the autistic population.

The third key finding of the current study is that middle-aged and older adults with high autistic traits report higher rates of suicidal self-harm compared to those with low autistic traits (13% vs 3%). Higher rates of progression from suicidal ideation to suicidal self-harm were also found in those with high autistic traits group versus the comparison group (18% vs. 8%). However, comparable progression rates from deliberate self-harm to suicidal self-harm were found in our high autistic and low autistic trait groups (64% vs 53%). The rates of suicidal self-harm documented in the current study are comparable to those found in the Swedish population registers documented in Hirvikoski et al. (2020) when taking into account the 70:30 female:male ratio in the PROTECT cohort; Hirvikoski et al. (2020) report that 8.4% of their autistic sample have experienced suicidal self-harm (autistic men = 5.8%, autistic women = 13.9%) compared to 12.7% of our high autistic trait sample (high trait men = 7.8%, high trait women = 15.1%). Similar rates of suicidal self-harm are also found in the comparison groups in Hirvikoski et al. (2020) and the current study (comparison men = 1.4%, comparison women = 3.0%). While no sex differences were found in the current study, the higher rates of suicidal self-harm reported by autistic/high autistic trait women is a matter of great concern. As autism is often framed around stereotypical male behaviours, women often experience barriers to diagnosis (Bargiela et al., 2016; Crane et al., 2018; Leedham et al., 2020). Autistic women who were diagnosed as adults often describe the need to camouflage their autistic characteristics in social situations, which could lead to the experience of burnout (Leedham et al., 2020). Additionally, some autistic women experience difficulties with acclimating their identity as an autistic person with their feminine identity (Bargiela et al., 2016). These experiences could result in elevated stress and psychological distress, resulting an increased likelihood of poor mental health. As such, targeted interventions and specialised mental health support for people, particularly women, on the autism spectrum is needed, which help mitigate this risk for self-harm and suicidality.

The final key finding of the current study was that comparable rates of help-seeking behaviours were found in both high autistic trait and comparison groups after an episode of self-harm. Approximately 70% of those who had self-harmed went on to seek support from a healthcare practitioner, from friends or family, or a suicide helpline. However, the current study does not examine the efficacy of the support given after the self-harm incident. While it is reassuring that the majority of those who had self-harmed sought support, this similarity in help-seeking behaviours was unexpected. It has been well documented that people on the autism spectrum often experience difficulties with accessing general healthcare (Mason et al., 2019a), as well as care specifically for their mental health or experiences with self-harm and suicidality (Camm-Crosbie et al., 2018). These difficulties in healthcare access are often attributed to the support provided not taking into account additional conditions that the autistic person may have. Additionally, the healthcare support is often not tailored towards neurodivergent populations, and not optimally relevant or appropriate for an autistic person (Mason et al., 2019a). This argues for increased awareness of autism in existing support services, as well as the creation of autism-specific support services. Doing so would be a step towards providing appropriate healthcare and support to people on the autism spectrum, which in turn could address the high rates of poor mental health and suicidality widely documented in autistic populations.

When contextualizing the findings of this study, it is important to consider limitations, as well as strengths. A strength of PROTECT is its use of an online platform, allowing large scale recruitment from a wide geographical spread across the UK. However, use of self-report alone is a limitation, and collecting health care records and multiple informant measures will be important in future work. Additionally, due to the phrasing of the questions in the PROTECT study, it is not possible to differentiate between deliberate non-suicidal self-harm (i.e., self-harm without the intention of suicide) and suicidal self-harm (i.e., self-harm with the intention of suicide) in some questions, e.g., “Q3. Have you deliberately harmed yourself, whether or not you meant to end your life?”. Furthermore, as the PROTECT study only uses self-report measures, we are unable account for those who did not wish to disclose self-harm or suicidal behaviours. The self-report design in PROTECT (rather than using population registers or healthcare records) means that we are also unable to account for those who may have died by suicide. The demographic questionnaire used by PROTECT also only asks about sex (rather than gender identity), thus the experiences of minority gender identities (e.g., transmen, transwomen, non-binary people, etc.) are not able to be explored in this current sample. Another important point to consider is that older adults who engage in medical research are typically those who are physically and mentally able, which may lead to sampling biases, survivor effects, and poor generalizability of findings (Golomb et al., 2012). PROTECT is predominately female (~ 67%) and well-educated, which emphasises this point on generalizability. Finally, the criteria used to identify the AST group was a short, bespoke (albeit validated) set of questions rather than a standardized measure, and participants may have scored highly for reasons other than autism-related traits. Whilst these factors may limit the overall generalizability of the findings, the results still provide important new information about self-reported experiences of self-harm and suicidality in relation to socio-communicative abilities in a large population of older adults, and represent a first step towards greater understanding of aging for those with persistent poor socio-communicative functioning.

In conclusion, our study exploring self-harm and suicidality experiences of middle-aged and older adults suggests that those who self-report high autistic traits are at greater risk of experiencing suicidal ideation, deliberate self-harm and suicidal self-harm. These differences between older adults with high versus no autistic traits were also found to persist when taking into account current symptoms of depression. The high rates documented in the current study were mostly consistent with the previous literature. These findings taken together suggest that people on the autism spectrum, including those who do not have an autism diagnosis but nonetheless have high autistic traits, may benefit from targeted interventions to mitigate this vulnerability to self-harm and suicide. Furthermore, as mental health difficulties are often a precursor to self-harming behaviours, specialised mental health support for autistic populations could prevent periods of crisis that may lead to the escalation of suicidal ideation and self-harm to suicide. Further research is required to examine the pathways and mechanisms that underpin the high rates of self-harm and suicidality found in autistic/high autistic trait populations, as well as the identification of protective factors that increase resilience to periods of crisis.

## Supplementary Information

Below is the link to the electronic supplementary material.

Supplementary file1 (DOCX 32 kb)
