# Supplementary material for: Self-harm and Suicidality Experiences of Middle-Age and Older Adults With vs. Without High Autistic Traits
Source: J Autism Dev Disord. 2022 May 26;53(8):3034–46. doi: 10.1007/s10803-022-05595-y (PMC10313542; doi:10.1007/s10803-022-05595-y)
Supplement: Supplementary file 1 — Supplementary file1 (DOCX 32 kb) [file 10803_2022_5595_MOESM1_ESM.docx]

**Supplementary Materials**

**PROTECT Self-harm and Suicide questionnaire**

1. Many people have thoughts that life is not worth living. Have you felt that way?
   1. *No*
   2. *Yes, once*
   3. *Yes, more than once*
2. Have you contemplated harming yourself, for example by cutting, biting, hitting yourself, taking an overdose?
   1. *No*
   2. *Yes, once*
   3. *Yes, more than once*
3. Have you deliberately harmed yourself, whether or not you meant to end your life?
   1. *No*
   2. *Yes*
4. How many times have you harmed yourself?
   1. *Never*
   2. *Once*
   3. *Twice*
   4. *Three or more times*
5. Have you done any of the following to harm or endanger yourself? (check all that apply)
   1. *Self-injury such as self-cutting, scratching or hitting, etc.*
   2. *Ingesting a medication in excess of the normal dose*
   3. *Ingesting alcohol or a recreational or illicit drug*
   4. *Swallowing dangerous objects or products*
   5. *Stopping prescribed medication*
   6. *None of the above*
6. Have you deliberately taken an overdose or harmed yourself with the intention to end your life?
   1. *No*
   2. *Yes*
7. Following any time when you took an overdose or deliberately tried to harm yourself did you… (check all that apply)
   1. *Need hospital treatment (eg A&E)*
   2. *See anyone from psychiatric or mental health services, including liaison services*
   3. *See your GP*
   4. *Receive help from friends / family / neighbours*
   5. *Use a helpline / voluntary organization*
   6. *None of the above*

| *Supplementary Table 1. Demographic characteristics of the COA and AST groups by sex.* | | | | | | | |
| --- | --- | --- | --- | --- | --- | --- | --- |
|  |  | **COA Male (n = 3,200)** | **COA Female (n = 7,295)** | **AST Male (n = 90)** | **AST Female (n = 186)** | **Trait Difference** | **Gender Difference** |
| **Age** | *M (SD)* | 65.80 (7.04) | 60.94 (5.93) | 64.27 (7.43) | 62.34 (6.30) | F(1,10769) = 1.83, *p =* .176 | F(1,10769) = 68.39, *p* < .001***  Trait x Gender Interaction: F(1,10769) = 12.81, *p* < .001*** |
|  | *[95% CI]* | [65.55-66.04] | [60.80-61.08] | [62.71-65.82] | [61.43-63.26] |  |  |
|  | *Range* | 50 - 81 | 50 - 81 | 51 - 81 | 52 - 81 |  |  |
| **Marital Status** | *Married* | 2561 (80.2%) | 4955 (68.1%) | 65 (72.2%) | 10 (57.0%) | χ2 = 22.78, *p* < .001*** | COA: χ2 = 165.38, *p* < .001*** AST: χ2 = 15.36, *p* = .008** |
|  | *Widowed* | 105 (3.3%) | 371 (5.1%) | 2 (2.2%) | 6 (3.2%) |  |  |
|  | *Separated* | 33 (1.0%) | 144 (2.0%) | 3 (3.3%) | 2 (1.1%) |  |  |
|  | *Divorced* | 197 (6.2%) | 794 (10.9%) | 4 (4.4%) | 33 (17.7%) |  |  |
|  | *Civil Partner* | 15 (0.5%) | 41 (0.6%) | 0 | 1 (0.5%) |  |  |
|  | *Co-habiting* | 166 (5.2%) | 518 (7.1%) | 11 (12.2%) | 17 (9.1%) |  |  |
|  | *Single* | 117 (3.7%) | 453 (6.2%) | 5 (5.6%) | 21 (11.3%) |  |  |
| **Education history** | *School to 16* | 476 (14.9%) | 969 (13.3%) | 19 (21.1%) | 24 (12.9%) | χ2 = 2.97, *p* = .398 | COA: χ2 = 19.27, *p* < .001*** |
|  | *School to 18* | 947 (29.6%) | 2316 (31.8%) | 20 (22.2%) | 53 (28.5%) |  |  |
|  | *Undergraduate* | 1038 (32.5%) | 2528 (34.7%) | 32 (35.6%) | 67 (36.0%) |  |  |
|  | *Postgraduate* | 733 (22.9%) | 1463 (20.1%) | 19 (21.1%) | 42 (22.6%) |  |  |
| **Current employment status** | *Employed* | 1360 (42.6%) | 4449 (61.1%) | 44 (48.9%) | 96 (51.6%) | χ2 = 4.72, *p =* .094 | COA: χ2 = 431.21, *p* < .001*** |
|  | *Retired* | 1793 (56.1%) | 2538 (34.9%) | 43 (47.8%) | 79 (42.5%) |  |  |
|  | *Unemployed* | 41 (1.3%) | 289 (4.0%) | 3 (3.3%) | 11 (5.9%) |  |  |
| Note: Only significant gender differences are reported. * *p* < .05, ** *p* < .01, *** *p* < .001 | | | | | | | |

| *Supplementary Materials Table 2. Self-reported prevalence rates of self-harm and suicidal ideation in COA and AST groups below PHQ-8 depression cut-off.* | | | | | | | | | |
| --- | --- | --- | --- | --- | --- | --- | --- | --- | --- |
|  |  | **Control Older Adults (COA; n=10,195)** | | **AS Traits (AST; n=218)** | | **Group Difference** | **Effect Size (Cohen's *d*)** | **Odds Ratio** |  |
| **Many people have thoughts that life is not worth living. Have you felt that way?** | *No* | 7271 | (71.8%) | 66 | (30.4%) | χ2 = 272.20 *p* < .001*** | 1.11 [0.97-1.24] | 5.82ǂ [4.34-7.80] |  |
|  | *Yes, once* | 1504 | (14.8%) | 39 | (18.0%) |  |  |  |  |
|  | *Yes, more than once* | 1353 | (13.4%) | 112 | (51.6%) |  |  |  |  |
| **Have you contemplated harming yourself? E.g. by cutting, biting, hitting yourself, taking an overdose.** | *No* | 8667 | (85.3%) | 118 | (54.6%) | χ2 = 222.65 *p* < .001*** | 1.01 [0.87-1.14] | 4.83ǂ [3.58-6.35] |  |
|  | *Yes, once* | 892 | (8.8%) | 34 | (15.7%) |  |  |  |  |
|  | *Yes, more than once* | 596 | (5.9%) | 64 | (29.6%) |  |  |  |  |
| **Have you deliberately harmed yourself whether or not you meant to end your life?** | *No* | 9704 | (95.6%) | 178 | (82.8%) | χ2 = 77.31 *p* < .001*** | 0.61 [0.47-0.74] | 4.50 [3.12-6.49] |  |
|  | *Yes* | 448 | (4.4%) | 37 | (17.2%) |  |  |  |  |
| **How many times have you harmed yourself?** | *Never* | 9744 | (95.7%) | 181 | (83.4%) | χ2 = 90.30 *p* < .001*** | 0.65 [0.52-0.78] | 4.47ǂ [3.08-6.46] |  |
|  | *Once* | 256 | (2.5%) | 15 | (6.9%) |  |  |  |  |
|  | *Twice* | 79 | (0.8%) | 8 | (3.7%) |  |  |  |  |
|  | *Three or more times* | 99 | (1.0%) | 13 | (6.0%) |  |  |  |  |
| **Have you deliberately harmed yourself with the intention to end your life?** | *No* | 9951 | (97.7%) | 195 | (89.4%) | χ2 = 59.97 *p* < .001*** | 0.53 [0.39-0.66] | 4.99 [3.18-7.84] |  |
|  | *Yes* | 235 | (2.3%) | 23 | (10.6%) |  |  |  |  |
| Note: Self-harm and suicidal thoughts and behaviours measured using a bespoke measure. ǂ Odds ratio calculated with yes/frequency options collapsed. * *p* < .05, ** *p* < .01, *** *p* < .001 | | | | | | | | | |

| *Supplementary Materials Table 3. Self-reported prevalence rates of self-harm and suicidal ideation in COA and AST groups above PHQ-8 depression cut-off.* | | | | | | | | | |
| --- | --- | --- | --- | --- | --- | --- | --- | --- | --- |
|  |  | **Control Older Adults (COA; n=300)** | | **AS Traits (AST; n=58)** | | **Group Difference** | **Effect Size (Cohen's *d*)** | **Odds Ratio** |  |
| **Many people have thoughts that life is not worth living. Have you felt that way?** | *No* | 99 | (33.2%) | 9 | (15.5%) | χ2 = 8.05 *p* = .018* | 0.41 [0.12-0.69] | 2.71ǂ [1.28-5.74] |  |
|  | *Yes, once* | 39 | (13.1%) | 7 | (12.1%) |  |  |  |  |
|  | *Yes, more than once* | 160 | (53.7%) | 42 | (72.4%) |  |  |  |  |
| **Have you contemplated harming yourself? E.g. by cutting, biting, hitting yourself, taking an overdose.** | *No* | 173 | (58.4%) | 24 | (41.4%) | χ2 = 7.45 *p* = .024* | 0.38 [0.11-0.67] | 1.99ǂ [1.12-3.52] |  |
|  | *Yes, once* | 34 | (11.5%) | 6 | (10.3%) |  |  |  |  |
|  | *Yes, more than once* | 89 | (30.1%) | 28 | (48.3%) |  |  |  |  |
| **Have you deliberately harmed yourself whether or not you meant to end your life?** | *No* | 252 | (85.1%) | 40 | (70.2%) | χ2 = 7.48 *p* = .006** | 0.40 [0.11-0.68] | 2.43 [1.27-4.67] |  |
|  | *Yes* | 44 | (14.9%) | 17 | (29.8%) |  |  |  |  |
| **How many times have you harmed yourself?** | *Never* | 256 | (86.2%) | 41 | (70.7%) | χ2 = 8.88 *p* = .031* | 0.36 [0.07-0.64] | 2.59ǂ [1.34-4.98] |  |
|  | *Once* | 10 | (3.4%) | 5 | (8.6%) |  |  |  |  |
|  | *Twice* | 9 | (3.0%) | 4 | (6.9%) |  |  |  |  |
|  | *Three or more times* | 22 | (7.4%) | 8 | (13.8%) |  |  |  |  |
| **Have you deliberately harmed yourself with the intention to end your life?** | *No* | 272 | (90.7%) | 46 | (79.3%) | χ2 = 6.32 *p* = .012* | 0.36 [0.08-0.64] | 2.53 [1.20-5.34] |  |
|  | *Yes* | 28 | (9.3%) | 12 | (20.7%) |  |  |  |  |
| Note: Self-harm and suicidal thoughts and behaviours measured using a bespoke measure. ǂ Odds ratio calculated with yes/frequency options collapsed. * *p* < .05, ** *p* < .01, *** *p* < .001 | | | | | | | | | |
